# Pancreatic Cancer Related Health Disparities: A Commentary

**DOI:** 10.3390/cancers10070235

**Published:** 2018-07-18

**Authors:** Lisa Scarton, Saunjoo Yoon, Sungho Oh, Edward Agyare, Jose Trevino, Bo Han, Eunsook Lee, Veronica Wendy Setiawan, Jennifer B. Permuth, Thomas D. Schmittgen, Folakemi G. Odedina, Diana J. Wilkie

**Affiliations:** 1 Department of Family, Community and Health System Science, 1225 Center Drive, P. O. Box 100187, Gainesville, FL 32610, USA; lscarton@ufl.edu; 2 Department of Biobehavioral Nursing Science, College of Nursing, University of Florida, Gainesville, FL 32610, USA; yoon@UFL.EDU (S.Y.); sungho.oh@ufl.edu (S.O.); diwilkie@ufl.edu (D.J.W.); 3 Department of Pharmaceutics, College of Pharmacy & Pharmaceutical Sciences, Florida Agricultural and Mechanical University, Tallahassee, FL 32301, USA; edward.agyare@famu.edu; 4 Department of Surgery, College of Medicine, University of Florida, Gainesville, FL 32610, USA; 5 Department Surgery, Keck School of Medicine, University of Southern California, Los Angeles, CA 90033, USA; bohan@usc.edu; 6 Department of Pharmacology and Toxicology, College of Pharmacy & Pharmaceutical Sciences, Florida Agricultural and Mechanical University, Tallahassee, FL 32301, USA; eunsook.lee@famu.edu; 7 Department of Preventive Medicine, Keck School of Medicine, University of Southern California, Los Angeles, CA 90033, USA; vsetiawa@med.usc.edu; 8 Departments of Cancer Epidemiology and Gastroinestinal Oncology, Moffitt Cancer Center, Tampa, FL 33612, USA; Jenny.Permuth@moffitt.org; 9 Department of Pharmaceutics, College of Pharmacy, University of Florida, Gainesville, FL 32610, USA; TSchmittgen@cop.ufl.edu; 10 Department of Pharmacotherapy and Translational Research, College of Pharmacy, University of Florida, Gainesville, FL 32610, USA; FOdedina@cop.ufl.edu

**Keywords:** pancreatic cancer, racial disparities

## Abstract

We summarize the risk factors that may significantly contribute to racial disparities in pancreatic cancer, which is now the third leading cause of cancer deaths and projected to be second around 2030 in 12 years. For decades, the incidence rate of pancreatic cancer among Blacks has been 30% to 70% higher than other racial groups in the United States and the 5-year survival rate is approximately 5%. Diabetes and obesity have been identified as potentially predisposing factors to pancreatic cancer and both are more common among Blacks. Smoking continues to be one of the most important risk factors for pancreatic cancer and smoking rates are higher among Blacks compared to other racial groups. The overall risk of pancreatic cancer due to changes in DNA is thought to be the same for most racial groups; however, DNA methylation levels have been observed to be significantly different between Blacks and Whites. This finding may underlie the racial disparities in pancreatic cancer. Identification and prevention of these factors may be effective strategies to reduce the high incidence and mortality rates for pancreatic cancer among Blacks.

## 1. Introduction

Pancreatic cancer is a worldwide problem. It is the third leading cause of cancer death in the United States [1] and is expected to become the second most lethal cancer around 2030 [2]. The CONCORD-3 study of global cancer survival among 37.5 million patients diagnosed with cancer between 2000 and 2014 revealed that the 5-year survival trends for pancreatic cancer continue to remain low in all countries (5–15%) [3]. Although some racial groups have higher prevalence rates of known risk factors such as obesity, diabetes and tobacco use, these risks do not fully explain the increased incidence and mortality rates in these groups [4]. The purpose of this commentary is to highlight (1) the current trends in pancreatic cancer incidence, mortality and survival rates in Black and White populations, and (2) innovative directions for research into factors potentially contributing to these pancreatic cancer disparities.

## 2. Pancreatic Cancer Statistics

The Surveillance, Epidemiology and End Results (SEER) program by the National Cancer Institute provided statistical information about cancer. The SEER data indicate that the mortality rate in pancreatic cancer has been relatively stable with the incidence rate increasing by 0.5% every year from 2004 through 2015 [5], and high mortality rates that may be associated with lack of early detection and poor therapeutic options [5].

**Figure 1:**
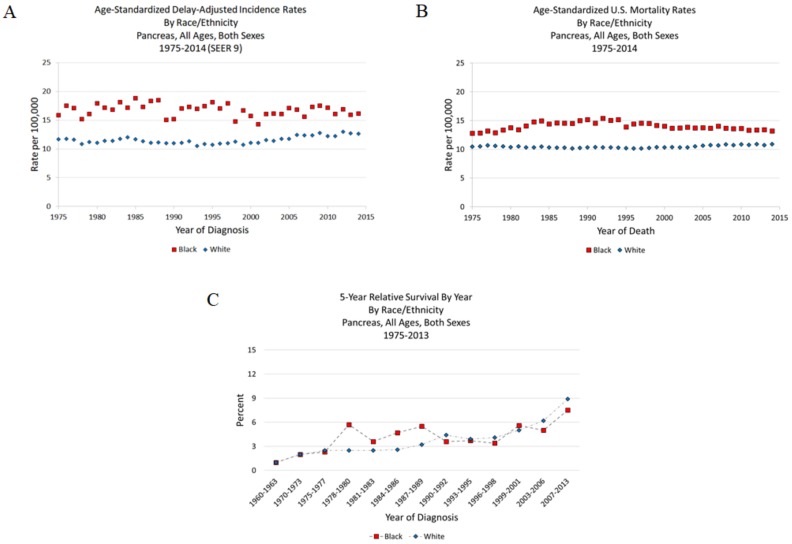
Pancreatic cancer statistics by race. (**A**) Age-Standardized Delay-Adjusted Incidence Rates. (**B**) Age-Standardized U.S. Mortality Rates. (**C**) 5-year Relative Survival by Year. All data for Blacks and Whites include Hispanic ethnicity.

For our analysis, we used SEER*Explorer data [6] (1975–2014) to estimate the incidence, mortality, and survival rates for Black and White Americans [7]. We did not exclude Hispanics, therefore, Black Hispanics and White Hispanics were included in the analysis. Figure 1A shows a clear gap between Blacks and Whites in incidence rates over time that is mirrored by the mortality rates over time (Figure 1B). These rates have been consistently higher for Blacks compared to Whites. Although this disparity seems to have narrowed in recent years compared to the early 90s, it is still prominent. The 5-year survival in Figure 1C shows moderate and steady improvement in survival for both races. Blacks are less likely to undergo surgery or chemotherapy compared to Whites [8,9,10], which may be associated with their uninsured status [9,11], their presentation at more advanced disease stage and, therefore, poorer expected survival [10].

Another analysis shows age-cohort, gender and race effects in pancreatic mortality rates. In an age-period-cohort-analysis of pancreatic cancer death rates for 1970–2009, the risk of death from pancreatic cancer was highest for the 1900 to 1910 birth cohort in men and the 1920 to 1930 birth cohort in women [12]. Furthermore, there was a statistically significant increase in period effects since the late 1990s in both White men and White women [12]. Increased period effects were also seen in the late 1990s among Black men and women, though not statistically significant, which is likely due to a small sample size. The worse disease prognosis for Blacks may be affected by known lifestyle factors in addition to sociocultural factors that have remained constant over time. Additionally, other factors are emerging as potential contributors that need additional research.

## 3. Socioeconomic and Lifestyle Factors as Contributors to Pancreatic Cancer Disparities

Socioeconomic and lifestyle factors are known contributors to pancreatic cancer health disparities. The role of these factors, however, in the increased incidence and mortality rates of pancreatic cancer in Blacks is still unknown.

### 3.1. Socioeconomic Factors

Socioeconomic status (SES) is associated with health disparities in cancer. For instance, SES affect person’s access to health care services, social and physical environments, behaviors and treatment. These factors apply to all cancers including pancreatic cancer [13]. In general, people who live in more deprived areas, have lower income and receive less education demonstrate higher incidence, mortality, and lower survival in different types of cancer [14]. SES and environmental factors are interconnected with bidirectional influence.

### 3.2. Lifestyle Factors

Known lifestyle risk factors for pancreatic cancer include obesity, diabetes, and tobacco use, which are associated with cancer incidence and mortality. However, findings are mixed on how these risk factors contribute to the racial disparities found in increased pancreatic cancer incidence and mortality rates among some racial groups, particularly Blacks [15].

Obesity is associated with an increased risk of pancreatic cancer. Although Black adults are 1.4 times more likely to be obese compared to their White counterparts [16], the role obesity plays in the increased incidence and mortality rates of pancreatic cancer is mixed. In a pooled analysis of 7 cohort studies, Bethea and colleagues [17] found Black participants who had a body mass index (BMI) between 30.0 and 34.9 had an associated increased risk for pancreatic cancer mortality (HR = 1.25; 95% CI, 0.99–1.57) compared to those with a BMI between 25.0 and 29.9 (HR = 1.08; 95% CI, 0.90–1.31) [9]. These findings indicate that obesity may be a contributing factor to pancreatic cancer, but it does not necessarily explain the disparities in the incidence or mortality rates for Blacks.

As the rate of obesity increases, so does the rate of diabetes. The burden of diabetes is higher in racial and ethnic minorities compared to Whites [18]. For example, American Indians have the highest prevalence of type 2 diabetes followed by Blacks who are at a 77% higher risk of developing diabetes than their White counterparts [18]. In addition, links have been made between diabetes and pancreatic cancer across all races. In a meta-analysis of 35 cohort studies, findings indicated that diabetes was associated with almost a two-fold increase in pancreatic cancer (RR = 1.94; 95% CI, 1.66–2.27) compared to the general population [19]. As with obesity, diabetes may be a contributing factor to pancreatic cancer but it does not fully explain the disparity in Blacks.

Tobacco use is a well-established risk factor for the development of cancer, which likely influences disparities with respect to all types of cancers [20]. Estimates based on the *Cancer Prevention Study II*, a United States-based prospective cohort study, suggest that at least 25% of deaths from cancers (bladder, esophagus, kidney, larynx, lip lung, oral cavity, pancreas, and pharynx) are attributable to smoking [21]. Additionally, approximately 45,000 Blacks die from smoking-related diseases each year in the United States, which surpasses all other causes of death in the Black community [22]. Ironically, compared with Whites, Black individuals smoke fewer cigarettes, but are more likely to smoke menthol cigarettes and cigarettes with higher tar yields, achieve higher net indices of smoke exposure, and may be at risk of greater physical dependence to tobacco [23]. All these data cumulatively result in greater morbidity and mortality rates from tobacco-related diseases in the Black community.

## 4. Biological Factors Potentially Contributing to Disparities

The established risk factors associated with pancreatic cancer in Blacks compared to Whites do not completely explain the increased incidence and mortality rates from the disease in Blacks. Since Blacks present with pancreatic cancer at a younger age [24] and demonstrate more aggressive disease at the time of diagnosis compared to their White counterparts [25], there may be other micronutrient deficiencies and genomic explanations for the differences among Blacks and Whites.

### 4.1. Zinc Deficiency Factor

Micronutrient deficiencies are associated with the development of many diseases [26,27]. The pancreas is both an endocrine and exocrine organ. Like other secretory tissues such as prostate and mammary glands, the pancreas has unusual zinc requirements in regulating its biological functions [28]. There is a growing body of information implicating that zinc dysregulation plays a significant role in the pathogenesis of pancreatic cancer [29,30]. Zinc metabolism is tightly regulated through the integration of zinc import, sequestration, and export mechanisms. Up-to-date, 10 zinc efflux and 14 zinc influx transporters have been identified and their roles have been under extensive investigation [31,32]. Even though the relationship between the expression of intra-cellular zinc level and pancreatic cancer mortality rate is unclear based on the data generated from prostate cancer studies [33,34,35], a plausible hypothesis is that disparity of zinc absorption under both genetic and epigenetic regulation in different populations may be responsible for the higher mortality rates in Blacks pancreatic cancer patients.

Zinc is an essential nutrient to all organisms and serves as a catalytic or structural cofactor for more than 300 proteins [36]. Zinc homeostasis is critically important for human health and its concentration is strictly regulated through membrane transporters among sub-cellular organelles. Accumulated evidence suggests that decreased zinc levels have been reported in pancreatic cancer [37,38].

There is a possibility that Africans may have genetically downregulated zinc absorption. Africa is a mineral rich continent and the zinc levels in the drinking water and diet are generally high [39]. If the Africans’ zinc absorption rates were the same as other racial groups, Africans might suffer from zinc toxicity. Therefore, Africans may have genetically downregulated their zinc absorption capacity to avoid high toxic zinc levels [39]. Using computer simulation, Engelken et al. [40] found extreme population differences in human zinc transporter ZIP4 expression in Sub-Saharan Africa, indicating genetic natural selection for the survival advantages among different populations. Just as European white people carry an evolutionary disadvantage against the solar UV light outside their low UV light ancestral environment [41], the low absorption capacity of zinc has created a disadvantage in people of African descent when they migrate outside Africa. Another ecologic study found an inverse relationship between soil zinc content and cancer rate [42], which provides indirect evidence implicating zinc regulation.

Zinc regulation may differ among modern human populations. The high mortality rates in Black pancreatic cancer patients indicate an imminent need to conduct further studies that represent Blacks who are susceptible to zinc deficiency and underrepresented in the current literature. Also, further studies are needed to examine the association between zinc transporter expression, basal intracellular zinc levels, and zinc uptake with the risk of pancreatic cancer prevalence and mortality. Such research could lead to intervention studies with zinc supplementation for pancreatic cancer prevention in Blacks. Clearly, much research is needed to confirm the role of zinc in pancreatic cancer.

### 4.2. Genomic Factors

The most common type of pancreatic cancer is exocrine cancer with 95% of exocrine cancers being pancreatic ductal adenocarcinoma (PDAC). Pancreatic cancer is commonly associated with activating mutations in the oncogene *KRAS* (mutated in over 90% of all PDAC cases) as well as mutations in the tumor suppressors *TP53*, *SMAD4* and *CDKN2A*/*p16* [43]. There are currently limited data on the genetic susceptibility of pancreatic cancer in non-Whites (recently reviewed [39]). Whether common genetic variants contribute to the observed disparities in pancreatic cancer susceptibility is unknown. Several susceptibility genes/loci for pancreatic cancer have been identified in previous genome-wide association studies (GWAS) [44]. Because the prevalence of the risk of alleles varies across populations, they could contribute to variation in pancreatic cancer susceptibility.

Recent findings suggest that the global DNA methylation at the epigenetic levels and its associated alteration in gene expression plays a critical role in the development of pancreatic cancer. This finding provides valuable insights for a better understanding of the initiation and progression of the disease. DNA methylation can lead to multiple abnormal cell biology including tumor grade, tumor stage, and patient’s length of survival time [45]. Strikingly, findings have been reported that DNA methylation changes of key cell-fate-determining genes are strongly associated with pancreatic cancer progression. Three genes, such as FAM150A, ONECUT1 and RASSF10, which exerted increased methylation in the promoter region, strongly correlate with decreased patient survival [46]. Particularly, ONECUT1, known as HNF6, plays a phenotypic switch in acinar-to-ductal metaplasia that can lead to PDAC [47]. It has been reported that about 23,000 CpG sites (Δβ ≥ 0.1) were differentially methylated between normal and tumor samples and the majority of the CpG sites were hypermethylated in pancreatic cancer [45].

The epidemiological data on racial differences in DNA methylation in pancreatic cancer development are not currently available. However, numerous reports indicate there are racial differences, particularly between Blacks and Whites, in the DNA methylation level in various cancer types including breast [48] and prostate [49] cancers. There is also a growing body of evidence on racial differences in DNA methylation alterations in normal tissue and during oncogenesis including the development of pancreatic cancer. It has been reported that DNA methylation levels are significantly different between Blacks and Whites at a subset of CpG dinucleotides associated with genes involved in oncogenesis at birth [50]. Since DNA methylation patterns play an unambiguous role in the pathways of cancers including pancreatic cancer, the fact that nearly 14% of the autosomal CpGs difference in DNA methylation between these two races provide ample evidence on the role of epigenetic levels in different races. This racial disparity in DNA methylation might affect the expression of adjacent genes involved in oncogenesis and could affect genetic response to physiological and environmental influences [36]. This finding suggests that the status of DNA methylation at pre-cancer stages may play a critical role in later racial differences in the incidence and mortality rates of pancreatic cancer. Further research is needed to understand how DNA methylation influences the development of pancreatic cancer across racial groups.

## 5. Conclusions

Currently, pancreatic cancer is the third leading cause of cancer deaths in the United States and projected to be the second in 12 years [1,2]. Blacks with pancreatic cancer have an overall worse prognosis when compared to their White counterparts, stage for stage, with a higher rate of incidence, a lower rate of receiving currently available treatment strategies, and a higher mortality rate [5,10,51]. Recognized socioeconomic and lifestyle risk factors independently do not fully explain the increased incidence and mortality rates in Blacks. The disparities may result from a multifactorial interplay of socioeconomic, lifestyle, micronutrient, and genomic factors but the exact impact of these factors remain unknown. Novel studies are needed to understand the role that zinc deficiency, genetic variation and DNA methylation at pre-cancer stages, which may play in later racial differences in the incidence, response to treatments, and rate of mortality of cancers. Research is also needed to better understand the impact of the multiple factors and tumor targeted treatments on racial disparities in pancreatic cancer.
